# Analysis of the Machinability of Copper Alloy Ampcoloy by WEDM

**DOI:** 10.3390/ma13040893

**Published:** 2020-02-17

**Authors:** Katerina Mouralova, Libor Benes, Tomas Prokes, Josef Bednar, Radim Zahradnicek, Robert Jankovych, Jiri Fries, Jakub Vontor

**Affiliations:** 1 Faculty of Mechanical Engineering, Brno University of Technology, 616 69 Brno, Czech Republic; tomas.prokes@vutbr.cz (T.P.); bednar@fme.vutbr.cz (J.B.); zahradnicek@vutbr.cz (R.Z.); jankovych@fme.vutbr.cz (R.J.); 184157@vutbr.cz (J.V.); 2 Faculty of Production Technologies and Management, Jan Evangelista Purkyně University, 400 96 Ústínad Labem, Czech Republic; benes@fvtm.ujep.cz; 3 Department of Production Machines and Design, Technical University of Ostrava, 708 33 Ostrava, Czech Republic; jiri.fries@vsb.cz

**Keywords:** WEDM, electrical discharge machining, ampcoloy, design of experiment, machining parameters

## Abstract

The unconventional technology of wire electrical discharge machining is widely used in all areas of industry. For this reason, there is always an effort for efficient machining at the lowest possible cost. For this purpose, the following comprehensive study has been carried out to optimize the machining of the copper alloy Ampcoloy 35, which is particularly useful in plastic injection moulds. Within the study, a half-factor experiment of 2^5-1^ with 10 axial points and seven central points of a total of 33 rounds was carried out, which was focused on the response monitoring of the input factors in the form of the machine parameters setup: gap voltage, pulse on time, pulse off time, discharge current, and wire speed. Based on the study of the response in the form of cutting speed and surface topography, their statistical models were created, while the optimal setting of machine parameters was determined to maximize the cutting speed and minimize the topography parameters. Further, a detailed cross-sectional analysis of surface and subsurface layer morphology was performed using electron microscopy including chemical composition analysis. In order to study microstructural changes in the material at the atomic level, a lamella was created, which was then studied using a transmission electron microscope.

## 1. Introduction

Electrical discharge machining (EDM) is an unconventional machining technology in which the material is collected by periodically repeating electrical pulses between the workpiece and the tool electrode in an environment of a usually liquid dielectric. The main advantage of this technology is the ability to machine materials after heat treatment with high hardness. Since no mechanical forces are applied between the tool and the workpiece, it is possible to machine even very soft materials without plastic deformation or thin-walled profiles. Thanks to the high precision and quality of machined surfaces, it is possible to machine even very complex shapes which are most often used in the production of pressing tools. However, the main drawbacks are relatively slow material removal and energy intensity [1,2].

Wire electrical discharge machining (WEDM) uses a wire of 0.3 to 0.02 mm in diameter, usually made of brass or copper, molybdenum or composite as the tool electrode [3]. This machining technology is widely used in all areas of industry, especially in the automotive, aerospace, military, and medical industries. It also enables micro-dimensional machining with very small dimensions and very good accuracy [4].

The WEDM technology is specific in terms of the morphology of machined surfaces, which are formed by a large number of craters. Similar craters can also be studied after the electrical discharge sinking [5] or micro electrical discharge sinking [6,7] of various materials. The mechanism of formation of these craters consists in the formation of individual electric discharges, which cause the material to erode in the form of tiny balls, which are subsequently washed away from the cutting point by a dielectric liquid. The simulations of single crater formation in WEDM have been performed by several authors, such as Han [8] or Giridharan [9]. In addition to the erosion itself, the workpiece material is also removed by evaporation due to very high temperatures at the cutting point, ranging from 10,000 to 20,000 °C [10]. At the same time, these very high temperatures cause intense diffusion processes between the short-term fully molten workpiece material and the wire electrode. When the material has been cooled, a recast layer is formed on the workpiece surface, which has a different thickness and also covers a different percentage of the surface. The thickness and percentage of the recast layer depend on many factors, from the machine parameters setting through the orientation of the semi-product to the type of material to be machined and its additional heat treatment, which was studied in Mouralova study [11] for X210Cr12 steel.

Ampco alloys find their application especially in moulds for plastic injection. Their advantages include high thermal conductivity up to 208 W·m^−1^·K^−1^ while achieving hardness up to 450 HB. Due to the high thermal conductivity, the injection moulding cycles can be reduced by approximately 20–80% compared to commonly used materials [12].

Thankanchan [13] focused on the evaluation of the machining characteristics of WEDM using Taguchi method and Grey relational analysis. They studied various WEDM processing parameters and material characteristics of Boron Nitride volume fractions. Afterwards, a mathematical model was developed which was based on the experimental values obtained for the material removal rate and the surface roughness, and it was proven to be precise to predict the output response. Nishida [14] carried out experiments to analyse a heat pipe with grooves produced by a copper tube using WEDM for the application in thermal management of electronic packaging. They tried to prove with the help of the experiments that the heat pipe works successfully while testing it horizontally to increasing heat loads. Bhuiyan [15] focused on the development of cooper based electrostatic micro actuator with the help of WEDM. During the experiments, it was found out that copper-based actuator design using WEDM technology was much simpler for batcher processing and could bring the advantages in rapid prototyping. Venkateswarlu [16] focused on experiments for the optimization of machining parameters, such as pulse on time, pulse off time, and wire tension in WEDM of copper, employing Taguchi analysis and establishing the regression equations between the process parameters and responses. According to the results obtained, the pulse on time proved to be the most significant factor affecting the material removal rate and surface roughness, which was followed by the pulse off time and wire tension. Ubale [17] aimed at modelling the process using the artificial neural network (ANN) employing WEDM of Tungsten-copper composite. They performed the experiments according to a central composite design approach of response surface methodology and developed different ANN models for the material removal rate. As a result, the predicted outcomes from ANN model were compared with the experimental values, which were satisfactory. Rao [18] studied and developed mathematical relations between the work piece thickness and other cutting parameters data for machining copper using CNC WDEDM. They focused on the effect of the work piece thickness on various machining parameters (discharge current, cutting speed and the material removal rate, spark gap). The outcomes of the experiments carried out were found to be encouraging and the mathematical equations could be applied for establishing the machine parameters and estimation of machining time.

WEDM technology is very difficult to optimize due to a large number of parameters entering the machining process, and the optimization is usually aimed at maximizing the cutting speed and the quality of the machined surface. Considering the number of input factors affecting the speed of cutting and the quality of the surface and subsurface layers, extensive research has been carried out on the effect of heat treatment on the occurrence of defects [11,19], the effect of section orientation by a semi-product [11,20] and the effect of the material used [21].

The purpose of the present study was to optimize the WEDM process in terms of the maximizing cutting speed and surface quality and to conduct a comprehensive study of the surface and subsurface area.The machining of the copper alloy Ampcoloy 35 by WEDM has not been studied in any study despite its ever-increasing use. Ampcoloy 35 has been studied for its widespread use in injection moulds.

## 2. Experimental Setup and Material

### 2.1. Experimental Material

**Figure 1:**
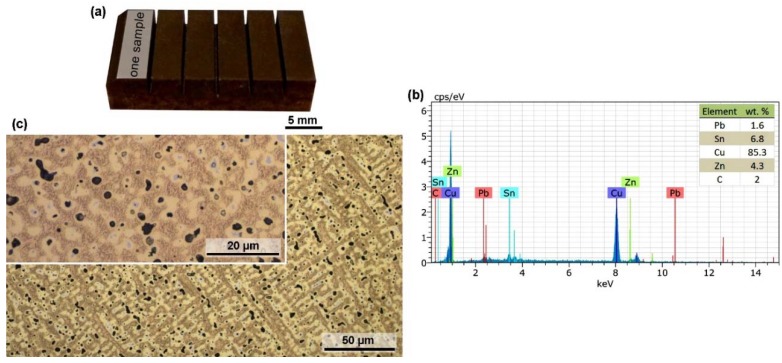
(**a**) Experimental samples; (**b**) Analysis of the chemical composition of the semi-product Ampcoloy 35; (**c**) Microstructure representation of the Ampcoloy 35 material.

Samples for the experiment were made of the copper alloy Ampcoloy 35, and this material has a chemical composition given by the EN standard in wt.%: 7% Sn, 4% Zn, 6% Pb and Cu-balance. Ampcoloy 35 contains a higher amount of tin, thus increasing the tensile strength to 250 MPa. Due to the low hardness value of 80 HB (10/30), coating technology is used in the field of plastic injection. Most often, a hydrogenated diamond-like carbon (H-DLC) coating is used, which is characterized by high hardness and at the same time supports heat dissipation from the melt. The alloys of the Ampcoloy series find their application primarily in the form of mandrels and complicated shaped parts, such as rib pressing inserts. A 10 mm prism semi-product was used for the experiment, with samples, microstructure and chemical composition analysis (EDX, Lyra 3, Tescan, Brno, Czech Republic) of the semi-product shown in Figure 1.

### 2.2. WEDM Machine Setup

For the production of samples, a WEDM cutter of the EU64 type from MAKINO (Meguro, Japan) was used. This machine is equipped with CNC control in all 5 axes, which allows for the production of conical shapes. The workpiece was immersed all the time in a dielectric bath containing unionized water. The instrument electrode was a 0.25-mm diameter wire made of brass (60% Cu and 40% Zn) supplied by PENTA (Prague, Czech Republic) with the marking PENTA CUT E.

The key output characteristics that describe EDM are the cutting speed and surface finish quality. The dependence of these characteristics on machine setting parameters was modelled using a design of experiment (DoE). For this purpose, the five most important adjustable independent parameters of the machine were selected: gap voltage (*U*), pulse on time (*T_on_*), pulse off time (*T_off_*), discharge current (*I*), wire speed (*v*), while the so-called factors and their experimental range was set: gap voltage min. 50 and max. 70 V, pulse on time min. 6 and max. 10 µs, pulse off time min. 30 and max. 50 µs, discharge current min. 25 and max. 35 A, wire speed min. 10 and max. 14 m·min^−1^.The limiting values of the individual parameter settings were determined based on the extensive previous tests and also with the recommendations of the machine manufacturer.

Within the design of the experiment, 33 partial experiments of the so-called runs were performed. These runs were arranged in a half factor experiment of 2^5-1^ with 10 axial points and 7 central points. This experimental plan was chosen primarily because it allows to model dependence on individual factors including quadratic curvature and their second-order interactions, and the statistical properties of this plan are described in detail in Montgomery [22]. The setting of input factors in individual partial experiments is described in Table 1.

### 2.3. Experimental Methods

The samples produced as part of a design of experiment on an EDM cutter were cleaned in an ultrasonic cleaner and subjected to complex analysis using a scanning electron microscope (SEM) LYRA3 from Tescan(Brno,Czech Republic). The part of this microscope was also an energy-dispersive X-ray detector (EDX), which enabled the analysis of the chemical composition. In order to study surface and subsurface layers, metallographic specimens were made which enabled a cross-sectional representation of the samples. The specimens were prepared by conventional techniques, namely wet grinding and diamond polishing using the automated preparation system TEGRAMIN 30 from Struers(Westlake, Cleveland, United States). The final mechanical-chemical polishing was carried out using an OP-Chem suspension from Struers. After the etching with aqua regia etch 1:20 (HCL:HNO_3_), the material structure was observed and documented using an inverted light microscope (LM) Axio Observer Z1m from ZEISS(Jena, Germany). The surface topography (2D surface maps), area, profile, and base profile parameters were studied using a non-contact 3D profilometer Taylor Hobson Talysurf CCI Lite. The measured data were then processed in TalyMap Gold software (5.1). 3D surface reliefs were also studied using the Atomic force microscopy (AFM) semicontact technique, and the measured data were analysed in Gwyddionprogramme. Using a focused ion beam (FIB) a Helios microscope from FEI(Hillsboro, OR, USA), a lamella was prepared to study the material composition using EDX in a transmission electron microscope (TEM) Titan from FEI.

## 3. Results and Discussion

### 3.1. The Statistical Evaluation of Surface Topography and Cutting Speed

The topography of machined surfaces must always comply with the values specified in the product documentation. Therefore, it should be carefully monitored to avoid the deterioration of topography beyond the prescribed values and hence the production of rejects. The analysis of the topography of the surface in relation to the setting of the machine parameters is therefore necessary, especially in cases where the part is machined only by WEDM cutter without further finishing operation, which is usually grinding. For this reason, three basic profile parameters, three profile parameters, and three of their area equivalents were evaluated in the experiment. The evaluated parameters of the basic profile were Pa, Pz, and Pq. The parameters evaluated by the profile method were Ra, Rz and Rq. The parameters Sa, Sz, and Sq were evaluated by an area method. All parameters were evaluated using a contactless profilometer Taylor Hobson according to the corresponding standard for area parameters ISO 25178-2 [23] and profile ISO 4287 [24]. All parameters were evaluated on 1024 profiles of a single evaluation length lr=0.8 mm obtained from S-F surfaces of the measurements made with 20× objective. Five random points on each sample were selected for the measurement and the average of these values was subsequently made.

**Figure 2:**
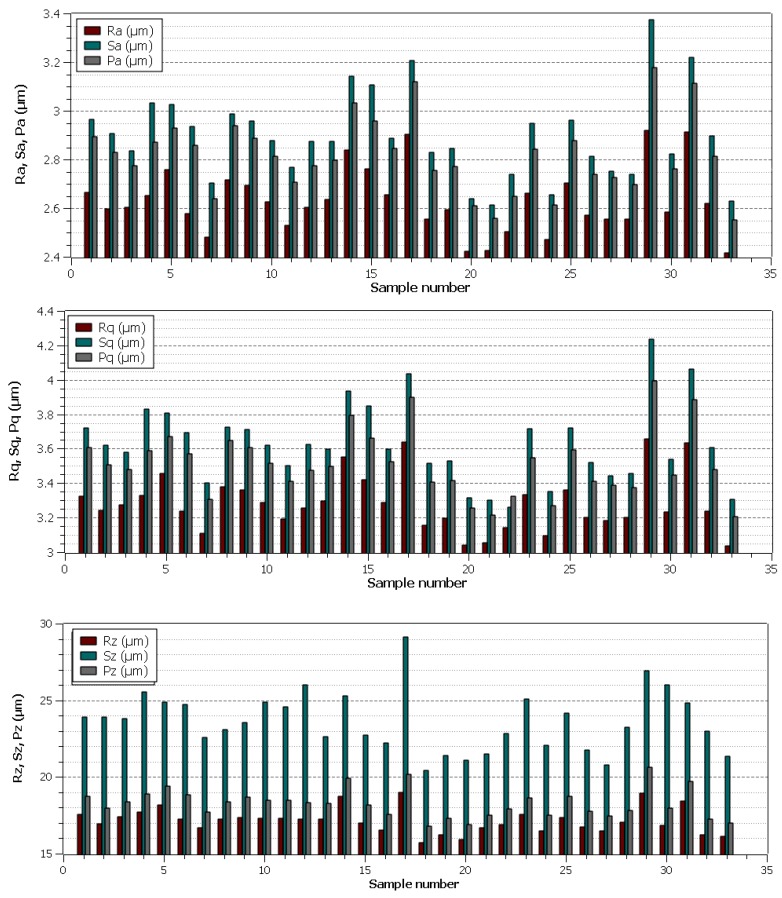
The evaluated basic profile parameters, profile and area parameters of individual experimental samples.

All nine evaluated topography parameters for 33 specimens are arranged in Figure 2, with correlations between these parameters being apparent. The lowest values of all parameters were achieved for Samples 20, 21, and 33 (Ra value of 2.4 µm), which were equally machined with the pulse on time parameter set to 6 µs and also with the same discharge current set to 25 A. Other three parameters were set differently. It can be stated in general, however, that even these lowest topography values are higher than in pure copper machining in the Venkateswarlu study [16], when Ra of 1.6 µm was achieved, but other topography parameters were not evaluated. However, this is the only study that has been involved in the machining of copper and its alloys using WEDM evaluation of the surface topography.

**Figure 3:**
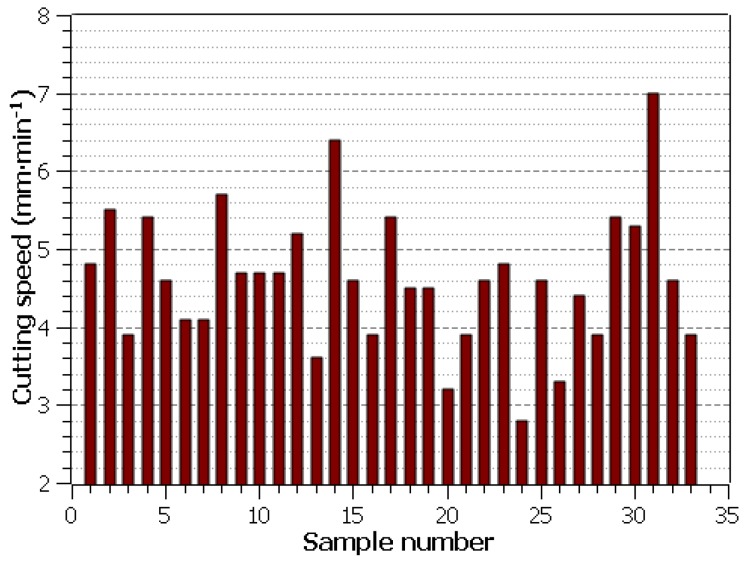
Thecutting speed of individual samples.

Compared to conventional technologies, WEDM is completely different in terms of speed machine control. It does not allow direct adjustment of the cutting speed *v_c_*(mm·min^−1^) when programming the machine but is based on the setting of individual machine parameters, while the WEDM machine used enabled the direct speed measurement during the machining process. The section length of each sample was always 3 mm. The cutting speed read is shown in Figure 3 for each sample, wherein the wire electrode has never been broken. The highest cutting speed was achieved with machine settings: *U* = 50 V, *T_on_* = 10 µs, *T_off_* = 30 µs, *v* = 14 m·min^−1^ and *I* = 35 A for Sample 31, this speed was 7 mm·min^−1^.

Regression analysis was used to evaluate the experiment, where a full quadratic hierarchical model was selected with the selection of stepwise predictors at a significance level of 0.05. The analysis was performed in the statistical software Minitab 17. All predictors included in the model were significant or their interaction was significant and were included in the model because of the hierarchy.

Since all topography parameters show statistically significant Spearman correlations (*p*-value<0.0005), the most commonly used parameter Ra was chosen for modelling. However, the models for the other parameters would be similar. The following statistical response model was created for the cutting speed:

(1) $$\begin{array}{l} v_{c}=-0.84+0.0444U-0.4111T_{on}-0.0075T_{off}+0.1583v+0.16I+0.0005U\cdot T_{off}-\\ -0.002U\cdot I+0.0225T_{on}\cdot I-0.0031T_{off}\cdot v-0.0015T_{off}\cdot I, \end{array}$$

where *v_c_* (mm·min^−1^) is cutting speed and a model was created for the surface topography parameter Ra:

(2) $$Ra=1.538+0.0563T_{on}+0.0213I,$$

where Ra (µm) is arithmetical mean deviation of profile.

The determination coefficients that describe the percent variability of the measured data described by the model are 99.25% for the cutting speed and 76.68% for the topography parameter Ra. The parameter model Ra described about 77% of variability, which is relatively good for WEDM surface topography models, and the model contains only two linear terms.

**Figure 4:**
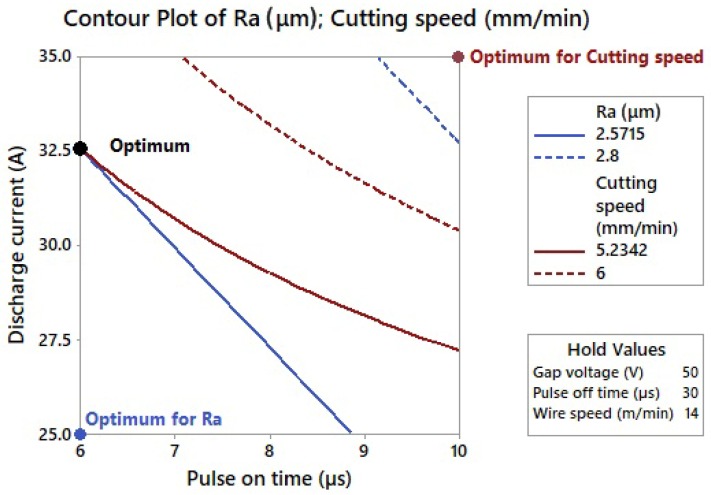
A multi-criteria optimization.

If the same significance is assigned to both responses and the cutting speed is required to be maximum and Ra—minimum, the optimal parameter settings are obtained by the multi-criteria optimization in Minitab 17: *U* = 50 V, *T_on_* = 6 µs, *T_off_* = 30 µs, *v* = 14 mm·min^−1^, and *I* = 32.5 A. With this parameter setting, the cutting speed would be 5.23 mm·min^−1^ and Ra—2.57 µm, as shown in Figure 4.

Since the topography parameter Ra is dependent only on two machine setting parameters, the response contour lines can be easily plotted at optimum. It is clear from the graph that the requirements for the maximum cutting speed and minimum Ra go against each other because the response surfaces have almost the same shape while minimizing one response and maximizing the other.

**Figure 5:**
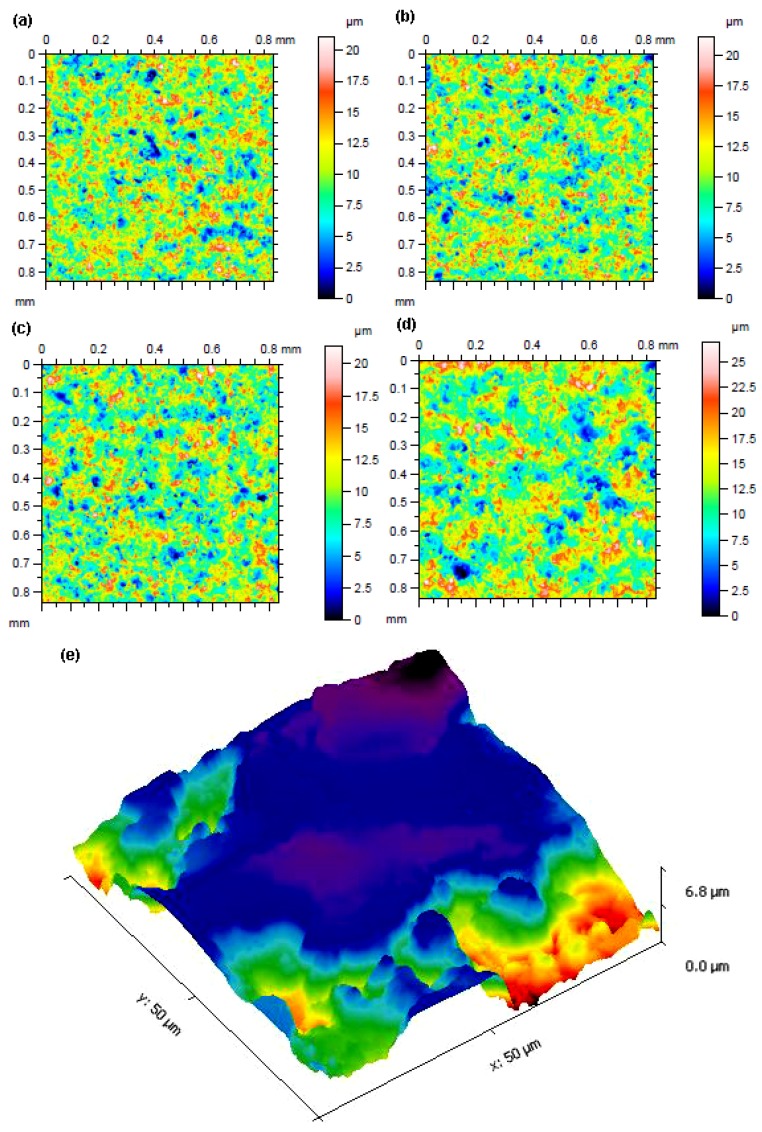
(**a**) 2D colour-filtered relief of Sample surface 20, (**b**) 2D colour-filtered relief of Sample surface 21, (**c**) 2D colour-filtered relief of Sample surface 33, (**d**) 2D colour-filtered relief of Sample surface 29, (**e**) 3D surface relief of Sample 20 obtained by AFM.

To facilitate easier visualization of the reliefs of machined surfaces, a 2D colour-filtered surface scan of three samples (20, 21 and 33 with Ra values of 2.4 µm) was created on a 3D profilometer Taylor Hobson Talysurf with the lowest surface topography values as well as one with the highest (29), i.e., with the worst surface quality. All these scans are shown in Figure 5a–d, whereby the colour filter clearly makes it possible to observe the height differences between the individual protrusions and depressions, which are always randomly distributed on the WEDMed surface, a non-periodic topographic surface. This non-periodic surface has the disadvantage of the difficulty of measuring topographic parameters. In contrast to conventional machining methods, where the relief is periodic, the measurements here need to be carried out very carefully and completely randomly at several different locations, which are chosen using the same principle. A semi-contact AFM technique was used to obtain 3D relief of the surface imaging, which is based on the detection of changes in the interaction forces between the tip and the workpiece surface with the change in the tip distance from the surface. The measurement was performed in Scanasyst mode using a tip with a radius of 0.65 µm. The area evaluated was 50×50 µm and is shown in Figure 5e, wherein the individual craters and their shape are apparent. The shape of the individual craters is different for different materials as well as their additional heat treatment, which was studied for the Ti-6Al-4V material, X210Cr12 and 16MnCr5 steels, and AlZn6Mg2Cu aluminium alloy [25].

### 3.2. The Analysis of Surface and Subsurface Area

The surface morphology of all machined samples was studied by electron microscopy. A backscattered electron detector (BSE, Lyra 3, Tescan, Brno, Czech Republic) was used for all imaging, with the samples always studied at 1000×, 2500×, and then 4000×.

**Figure 6:**
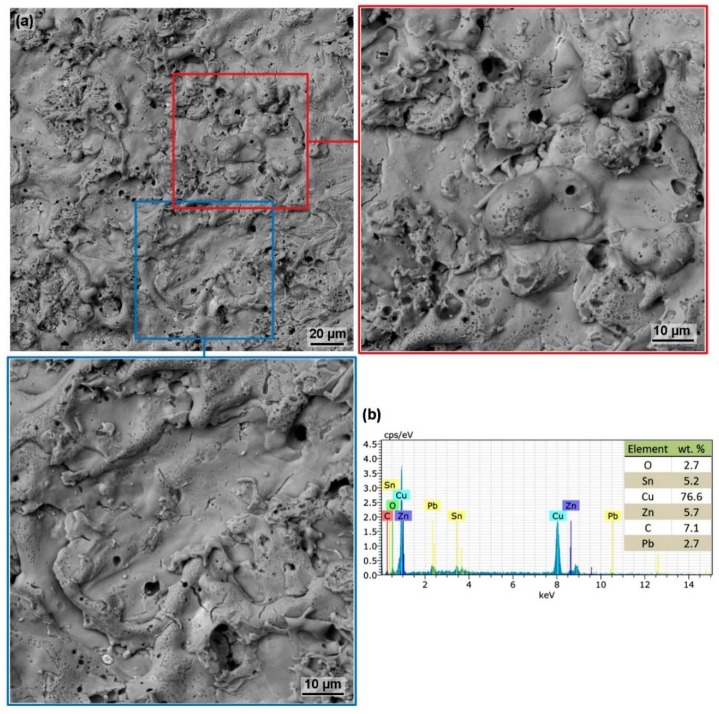
The surface morphology of Sample 20 with the lowest surface topography values machined with the parameters: *U* = 70 V, *T_on_* = 6 µs, *T_off_* = 50 µs, *v* = 14 m·min^−1^ and *I* = 25 A, SEM (BSE) including details of two points and the chemical composition analysis (**a**) sample surface, (**b**) chemical composition analysis from the entire area shown in image (**a**).

The morphology of all machined samples within the design of the experiment was similar, with no significant differences depending on the machine parameter settings. All samples were relatively smooth, as shown in Figure 6 with not very significant individual craters, as was the case with Inconel 625 [20]. On the surface of all samples, there were several small cracks, which were subsequently studied in a cross-section of the samples to determine their influence on the service life and correct functionality of the machined parts. The crack defects are a relatively common phenomenon in WEDM and have also been investigated for pure titanium [26], non-composite ceramics [27] or tungsten carbide [28]. In addition, there were a number of small craters up to 5 µm in diameter on the surfaces, which were probably formed as individual bubbles produced during the erosion process. The microstructure of the material in the form of dots is also noticeable in some places, which has also been studied in Inconel 625 [20]. The analysis of the chemical composition in the selected area of 200 × 200 µm, the resulting spectrum of which is shown in Figure 6b showed low contamination with a tool electrode element, zinc, which increased from 4 wt.% to 5.7 wt.%. Unfortunately, the diffusion of copper cannot be determined because the material to be processed was the copper alloy.

**Figure 7:**
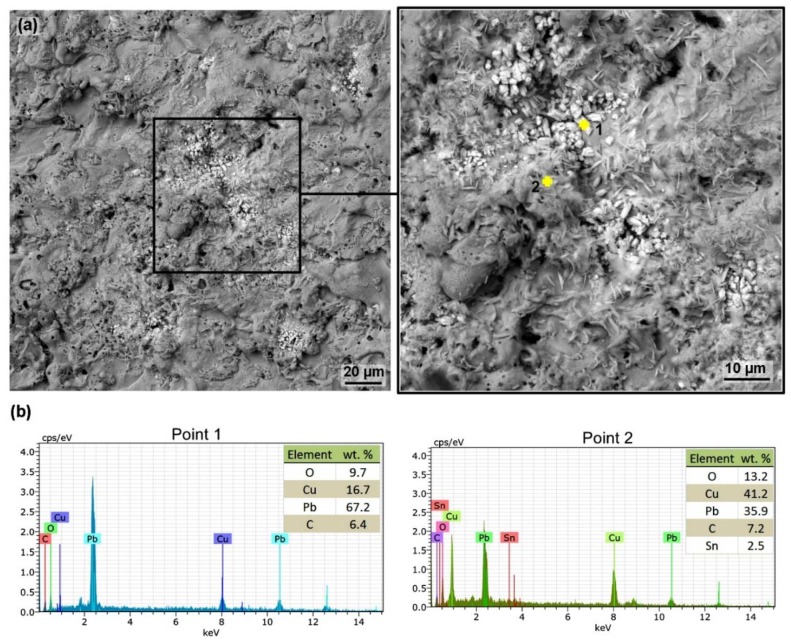
Surface morphology of Sample 20 with the lowest surface topography values machined with parameters: *U* = 70 V, *T_on_* = 6 µs, *T_off_* = 50 µs, *v* = 14 m·min^−1^ and *I* = 25 A, SEM (BSE) including the detail and chemical composition analysis at two points (**a**) sample surface, (**b**) chemical composition analysis at Points 1 and 2.

There were also locations on each surface of the machined samples with lead crystals segregated on the surface, an example of which is shown in Figure 7 including chemical composition analysis at two different points. The whiter the place was, the higher the lead content was. Lead crystals of this type have not been observed on any material after EDM, and this is a completely new phenomenon. This phenomenon is probably caused by the lead segregation due to a change in solubility. In liquid copper, the solubility of lead is higher than in solid copper, which during the cooling leads to the elimination of the excess lead in the form of crystals.

**Figure 8:**
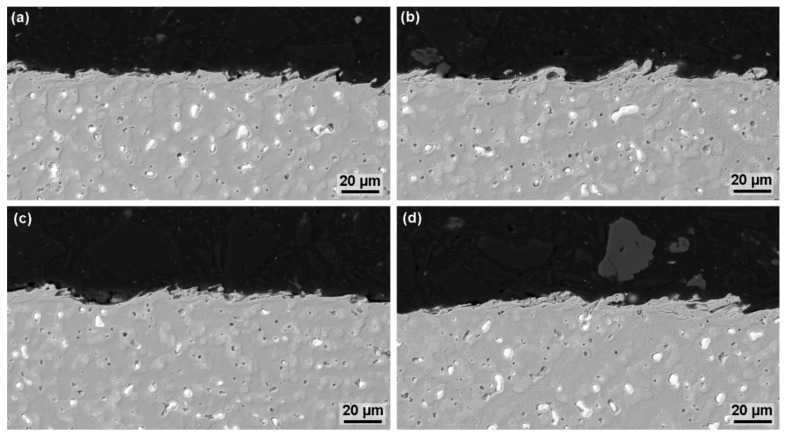
Cross-section of the samples, SEM (BSE) (**a**–**c**) Samples 20, 21 and 33 having the lowest Ra values of 2.4 µm, (**d**) Sample 29 with the worst surface quality in terms of topography.

The analysis of the subsurface layer was performed on pre-prepared metallographic preparations of all samples using electron microscopy. The knowledge of the state of the subsurface layer is a key aspect necessary to determine and assess the proper functionality of a manufactured component and its expected service life. In fact, if there are defects in the subsurface area in the form of cracks, such as X210Cr12 steel and their various heat treatments [11] or in the form of burnt cavities, such as Creusabro 4800 [29], Hardox 400, or Hadfield steel [21], it is very likely that the correct functionality or service life of a component will not be maintained. For this reason, it is always necessary to know whether it is necessary to optimize the machine setting parameters, the cutting direction of the semi-product or other aspects to eliminate these subsurface defects or not. A BSE detector was used throughout the whole analysis, first with a magnification of 1000× and then with a magnification of 2500× and 4000×, with no subsurface defect found on any of the samples produced. This is also shown in Figure 8, which shows cross sections of the three best samples and one worst, in terms of surface topography. The recast layer is relatively rugged and unrelated to thicknesses up to 15 µm, but does not contain any small cracks that have been studied from above. It can, therefore, be concluded that these tiny cracks on the surface are only a few micrometres deep and are therefore not noticeable at all in the cross-section.

### 3.3. TEM Lamella Analysis

**Figure 9:**
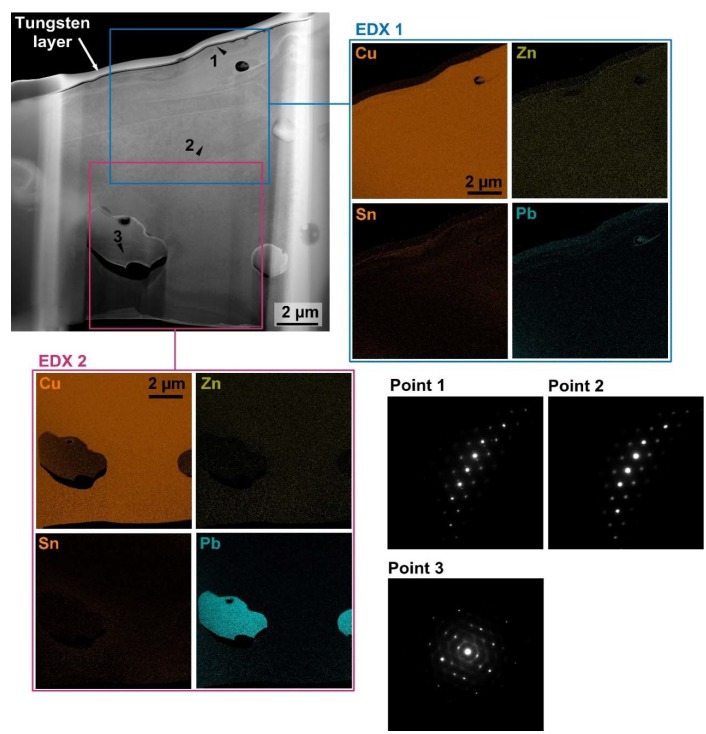
TEM lamella with two EDX measurement points and three other diffraction measurement points, Point 1—the recast layer point, Point 2—the base material point, Point 3—the lead particle.

The production of TEM lamella from the surface of the machined Sample 20, which had the lowest values of topography parameters, was performed by means of Helios electron microscope equipped with ion and electron beam. The lamella production itself consisted of deposition of a protective tungsten block and subsequent sputtering of the double trench around it. Furthermore, the lamella was undercut, fixed by means of nanomanipulator to the holder, on which the final thinning to 0.2 µm thickness took place. Due to the size of the prepared lamella, 10×10 µm, which is shown in Figure 9, a chemical composition analysis was performed at two points in the scan setup mode with an acceleration voltage of 300 kV and a current of 0.8 nA. EDX 1 was performed in the upper part of the lamella, which contained a protective layer of tungsten as well as a recast layer including a base material part. The analysis in this area showed an increased concentration of basic alloying elements (Sn, Pb, Zn) in the area affected by WEDM machining. This increased concentration is likely to be explained by the diffusion of elements from the base material into the heat affected area. In the EDX 2 measurement area, the workpiece base material was included, where the element distribution was equal, with the only exception of lead particles. The occurrence of these lead particles correlates with the results of the SEM cross-sectional analysis of the sample, which is shown in Figure 8. To determine the impact of WEDM on the copper alloy crystal structure, an additional diffraction mode measurement was performed in the heat-affected recast layer—Point 1 (close to the tungsten layer), compared to the base material measurement—Point 2, as shown in Figure 9. Comparison of diffraction patterns shows that in the recast layer (Point 1) there was a slight change in crystal orientation, which was reflected in the measurement by additional tiny bright points. This slight change in crystal orientation could be due to an increase in residual stress or recrystallization at the end of machining. In addition to determining the influence of the crystal orientation of the machined material as a result of WEDM, the crystal orientation was also determined for the lead particle, as shown in Figure 9. The diffraction pattern of the lead particle shows that it is a polycrystalline structure (light dots randomly spaced) composed of differently oriented crystal planes.

## 4. Conclusions

Based on a 33-round design of experiment, samples of Ampcoloy 35 copper alloy were produced to optimize the cutting speed, surface topography, and complex surface and subsurface analysis, with the following conclusions:

- the lowest values of all surface topography parameters were obtained for Samples 20, 21, and 33 (Ra value of 2.4 µm), which were equally machined with *T_on_* = 6 µs and also with the same *I* = 25 A,
- the highest cutting speed of 7 mm·min^−1^ was achieved for Sample 31 with the setting of machine parameters: *U* = 50 V, *T_on_* = 10 µs, *T_off_* = 30 µs, *v* = 14 m·min^−1^ and *I* = 35 A,
- a statistical response model was created for the topography parameter Ra, with all other parameters showing statistically significant Spearman correlations,
- a response model was created for the cutting speed, and after a subsequent optimization procedure where the equal significance was given to both responses and the cutting speed was required to be maximum and Ra minimum, we obtained the optimal parameter settings: *U* = 50 V, *T_on_* = 6 µs, *T_off_* = 30 µs, *v* = 14 mm·min^−1^ and *I* = 32.5 A, with this parameter setting the cutting speed would be 5.23 mm·min^−1^ and Ra 2.57 µm,
- the morphology of all machined samples was similar, with no significant differences depending on the setting of the machine parameters; the samples were relatively smooth with not too significant individual craters,
- there were several small cracks on the surface of all the samples but none were not found in cross-section, indicating their purely surface character, which did not affect the service life or functionality of the parts,
- all surfaces of the machined specimens have areas with segregated lead crystals,
- the subsurface area of all samples was completely defect-free, with the recast layer being no more than 15 µm thick and only locally,
- TEM lamella analysis allowed to detect an increased concentration of alloying elements in the recast layer and also detected a change in crystal orientation due to WEDM.

From the above conclusions it can be clearly stated that the WEDM of copper alloy Ampcoloy 35 does not create surface or subsurface defects limiting the correct functionality or service life of the manufactured parts. Therefore, every effort can be made to optimize the cutting speed depending on the surface topography in order to reduce energy costs of machining.

## Figures and Tables

**Table 1:** Used in the experiment.

Number of Sample	Gap Voltage (V)	Pulse on Time (µs)	Pulse off Time (µs)	Wire Speed (m·min^−1^)	Discharge Current (A)	Number of Sample	Gap Voltage (V)	Pulse on Time (µs)	Pulse off Time (µs)	Wire Speed (m·min^−1^)	Discharge Current (A)
**1**	70	8	40	12	30	**18**	60	8	40	12	30
**2**	60	8	30	12	30	**19**	60	8	40	12	30
**3**	60	8	40	12	25	**20**	70	6	50	14	25
**4**	60	10	40	12	30	**21**	50	6	30	14	25
**5**	50	8	40	12	30	**22**	60	8	40	12	30
**6**	60	8	50	12	30	**23**	70	10	30	14	25
**7**	60	6	40	12	30	**24**	50	6	50	10	25
**8**	60	8	40	12	35	**25**	60	8	40	12	30
**9**	60	8	40	10	30	**26**	50	10	50	14	25
**10**	60	8	40	14	30	**27**	50	10	30	10	25
**11**	60	8	40	12	30	**28**	50	6	50	14	35
**12**	50	6	30	10	35	**29**	50	10	50	10	35
**13**	70	10	50	10	25	**30**	70	6	30	14	35
**14**	70	10	30	10	35	**31**	50	10	30	14	35
**15**	60	8	40	12	30	**32**	60	8	40	12	30
**16**	70	6	50	10	35	**33**	70	6	30	10	25
**17**	70	10	50	14	35						
